# Radiotherapy after Conservative Surgery in Ductal Carcinoma In Situ of the Breast: A Review

**DOI:** 10.1155/2012/635404

**Published:** 2012-05-13

**Authors:** Maurizio Amichetti, Cristiana Vidali

**Affiliations:** ^1^ATreP, Agenzia Provinciale per la Protonterapia, Via Perini 181, 38122 Trento, Italy; ^2^S.C. di Radioterapia, Azienda Ospedaliero-Universitaria, Via della Pietà 19, 34129 Trieste, Italy

## Abstract

Several large prospective and retrospective studies have demonstrated excellent long-term outcomes after breast conservative treatment with radiation in invasive breast cancer. Breast-conserving surgery (BCS) followed by radiotherapy (RT) is an accepted management strategy for patients with DCIS. Adding radiation treatment after conservative surgery enables to reduce, without any significant risks, the rate of local recurrence (LR) by approximately 50% in retrospective and randomized clinical trials. As about 50% of LRs are invasive and have a negative psychological impact, minimizing recurrence is important. Local and local-regional recurrences after initial breast conservation treatment with radiation can be salvaged with high rates of survival and freedom from distant metastases.

## 1. Introduction

The term ductal carcinoma in situ (DCIS) encompasses a heterogeneous group of lesions with different biological potential and clinical behaviour [1]. 

There is no consensus regarding how to optimize the treatment for patients with DCIS: mastectomy cures almost all patients, but it is considered an overtreatment in many cases, particularly when they are small mammographic-detected lesions.

Most women are eligible for breast conservative surgery (BCS), a major decision is whether or not radiotherapy (RT) must follow surgical excision in order to control any microscopic residual disease. It is likely that not all patients with DCIS require RT following BCS; it is important to identify patients at high risk of recurrence or progression to invasive breast cancer who can benefit from RT. It is argued that RT should be used selectively, because of possible short- and long-term morbidity, and also because radiation fibrosis may hamper interpretation of follow-up mammograms.

Unfortunately, until now, the ability to select DCIS that is likely to recur or progress to invasive breast cancer is still limited, and there is a lack of level-1 evidence supporting the omission of adjuvant RT in selected low-risk cases, which could potentially be adequately treated by complete local excision.

Patients with DCIS treated with BCS may recur approximately in the same proportion either as DCIS or invasive breast cancer [2]. Efforts have continued to attempt to define the subsets of patients to whom we should offer the different treatment options for local treatment (surgical excision alone or plus radiation or mastectomy). Most of the DCIS patients are interested in breast conservative treatment and consequently a major decision is whether or not to add radiation treatment. Irrespective of whether a low-risk group can be identified, the role of radiation after wide excision of DCIS remains controversial.

The aim of this paper is to report and summarize the indications for RT after BCS and the results of retrospective, prospective randomized studies and meta-analyses on this issue.

## 2. Retrospective Studies

Published population-based retrospective mono-institutional and collaborative studies have demonstrated excellent long-term outcomes after BCS with RT.

Multiple observational studies report lower rates of recurrence (DCIS or invasive) for patients undergoing BCS + RT over BCS alone. All but one [3] of the observational studies show a lack of mortality benefit associated with BCS + RT compared with BCS alone.

Surveillance, Epidemiology, and End Results data have shown that still a substantial number of patients in the United States are treated with excision alone, without radiation [4].

An international collaborative multi-institutional study conducted by 10 Institutions in Europe and North America involving 1003 patients with mammographically detected DCIS has been published with long-term results [5]. Adjuvant tamoxifen was not used. The median followup was 8.5 years; the 15-year overall survival (OS) was 89%, and the 15-year cause-specific survival (CSS) was 98%.

The rate of any local recurrence (LR) (DCIS plus invasive) was 19%. Significant factors for LR were found to be the final pathologically positive margins and the patient's younger age. For the favorable subgroup of patients aged ≥50 years with negative margins, the 10-year rate of local failure was ≤8%.

A retrospective French collaborative study reported on 882 DCIS treated from 1985 to 1995 [6]. Mastectomy, BCS alone, and BCS + RT were performed, respectively, in 20%, 22%, and 58% of patients. Thus 515 women out of 705, who were treated with BCS, received adjuvant RT; a 10 Gy boost was given to 52% of them. Hormonal therapy was administered to 13.4% of the patients. The crude 7-year LR rate was 2% (mastectomy subgroup), 31% (BCS subgroup), and 13% (BCS + RT subgroup) (*P* < 0.0001). RT reduced the LR rate by 65% in all histological subgroups, especially in comedocarcinoma and mixed cribriform/papillary subgroups.

The most numerous single institution series was published by the William Beaumont Hospital (USA) in 2005 [7]. Between 1981 and 1999, 410 cases were treated; 367 were managed with BCS (54 with lumpectomy alone and 313 with adjuvant RT—median dose: 45 Gy). Of these 313 patients, 298 received also a boost with a median dose of 16 Gy. Thirty patients (8.2%) experienced an ipsilateral breast tumor recurrence after breast conservative treatment; 25/313 patients (8%) after RT, 5/54 (9.3%) after BCS alone, and 2/43 (4.7%) developed a chest wall recurrence after mastectomy. Ten-year rates of LR, CSS, and OS were similar after mastectomy and breast conservative treatment. Young age (<45 years), close/positive margins, no breast irradiation, and lower electron boost energies (≤9 MeV) were associated with a higher risk of LR.

Schouten van der Velden et al. [8] published the results of a multicenter dutch retrospective study on 798 women treated between 1989 and 2003, selected by the Tumor Registry of the Comprehensive Cancer Centre East Netherland. The 5-year recurrence-free survival (RFS) was 75% for BCS alone (237 patients) compared to 91% for BCS followed by RT (153 patients) and 99% for mastectomy (408 patients) (*P* < 0.01). Independent risk factors for LR were treatment strategy, symptomatically detected DCIS, and presence of comedo necrosis. Margin status reached statistical significance only for patients treated with BCS.

A very large retrospective experience is reported by the Van Nuys group [9]. Nine hundred and nine cases were reported, treated from 1971 to 2000; 326 of them underwent mastectomy, 237 BCS + RT, and 346 BCS alone. In the group of patients treated conservatively (583), the LR rate was 28% after BCS alone and 20% after BCS + RT (*P* = 0.06), with a median time to LR of 25 and 57 months, respectively (*P* < 0.01). It has to be noted that in the RT group there were more patients with “close” (<1 mm) margins (35% versus 19%), and the median followup was 36 months longer. After having observed the importance of several factors (grading and comedo necrosis, size, and margins), Silverstein et al. proposed the well-known Van Nuys Prognostic Index (VNPI) [10] based on a three-point score for each predictor. In 2003, they added age to their prognostic score [11], identifying three risk subgroups: ≤39 years of age (high score), 40–60 years of age (intermediate score), and ≥61 years of age (low score). To patients with a low score (from 4 to 6), conservative surgery alone was recommended, to those with an intermediate score (from 7 to 9) postsurgical RT, and to those with a high score (from 10 to 12) mastectomy, since the LR incidence at 5 years appeared too high (around 50%) with a BCS + RT treatment.

Although a simple, and apparently easy task, this score, based on retrospective analysis of Van Nuys case studies, has not been easily reproduced in clinical practice and has not been still validated in a prospective study [12, 13].

Another collaborative group in Italy published the data of 139 cases of DCIS treated with BCS + RT, with a median followup of 81 months [14]. Actuarial OS, CSS, and RFS at 10 years were 93%, 100%, and 86%. The same group reported on 112 cases of subclinical DCIS treated between 1982 and 1993 [15]. At a median followup of 66 months, 8 LRs were observed, with a 10-year actuarial CSS and RFS of 100% and 91%, respectively.

In a recent retrospective study of the same group, in which 586 patients treated with BCS + RT were analyzed, the risk of LR was found to be 9.6% at 10 years. The risk of LR with respect to a number of known prognostic parameters (age, tumour size, nuclear grade) was evaluated. Only age resulted to be a statistically significant prognostic factor in the univariate analysis (*P* = 0.0009). The actuarial 10-year OS and CSS were 98.5% and 99%, respectively (data submitted for publication).

### 2.1. Meta-Analysis of Retrospective Studies

In 1999, Boyages et al. [2] published a meta-analysis of the most important retrospective studies for DCIS, that had undergone different treatments: mastectomy (1574 cases), BCS alone (1148 cases), and BCS + RT (1452 cases). The meta-analysis revealed that the relapse incidence was of 22.5% after BCS alone (with 43% invasive LR), 8.9% after BCS + RT (with 50% invasive LR), and of 1.4% after mastectomy (with 76% invasive LR). Considering only the conservative treatment, the RT reduced the relative LR risk of at least 50%. The major advantage on local control was noted in cases of DCIS with necrosis, comedocarcinoma, high nuclear grade, and positive or “close” margins.

## 3. Randomized Clinical Trials

The impact of RT after conservative surgery in women with newly diagnosed DCIS has been analyzed in four prospective randomized clinical studies. The patients had undergone conservative surgery consistent of quadrantectomy, tumorectomy, or segmental mastectomy.

In three of these studies, a comparison was made between the results of BCS alone and BCS followed by breast irradiation (two arms of randomization). A 2 × 2 factorial design was used in the fourth study; the aim was to see the effectiveness of either adjuvant RT or hormonal therapy with Tamoxifen (TAM); allocation of patients could have happened for both treatments (RT and TAM) or just for one of the two, reserving the second as the only other choice.

The first study, the American NSABP-B-17 trial [16], randomized 818 cases of DCIS, between October 1985 and December 1990, 80.4% of which mammographically diagnosed (Table 1).

The protocol required histological negative margins; however, inking of excision margins and specimen radiography were not routinely used in that era. Thus, in a central pathology review on histopathologic specimen, uncertain or positive margins were found in 17% of the cases [17].

The cumulative incidence of ipsilateral events, with a median followup of 12 years, was of 31.7% for the control group, compared to 15.7% for the group with RT (*P* < 0.000005); 76% were true LR within the same quadrant. Considering the invasive LR, the incidence lowered from 16.8% (control group) to 7.7% (RT group) (*P* = 0.00001); while for DCIS LR, it lowered from 14.6% to 8.0% (*P* = 0.001). Neither the cumulative incidence of contralateral tumors nor the OS (*P* = 0.80) differed significantly between the two groups.

Recently, the long-term results of the NSABP B-17 and B-24 trials have been published [18]. In the NSABP B-17 study, with a median followup of 207 months, the significant contribution of RT in reducing the ipsilateral events is confirmed (Table 2).

The regional recurrence and the distant metastasis incidence are comparable in the two groups; also the contralateral tumor incidence results are very similar as well as mortality from breast carcinoma or other causes.

The evaluation of the predictive factors was referred to in a publication from 1999 [17], in which were analyzed the results of a centralized pathological revision of 623 trial cases (77% of all study cases). With a median followup of 8.5 years, in the multivariate analysis only comedo necrosis was found to be an independent predictive variable for LR. However, it was observed that within all prognostic subgroups, an overall benefit from the use of RT was maintained.

In the second study, the European EORTC-10853 [19], 1010 patients, treated between March 1986 and July 1996, were randomized; in 71% of the cases the initial diagnosis was exclusively mammographic (Table 1).

The arm with RT received a total dose of 50 Gy in 25 fractions; only 5% of the patients received an additional boost with a median dose of 10 Gy. Negative margins represented one of the inclusion criteria; however, a centralized pathologic revision of 863 of the 1010 randomized cases (85%) revealed that the margins were positive or “close” (≤1 mm) for 8.5% of the cases, and not known in 13.5% cases [20].

The LR rate, with a median followup of 10.5 years, was of 26% in the control arm, compared to 15% in the RT arm (*P* < 0.0001). In the RT group was noted a decrease in the risk of invasive LR and DCIS LR of 42% (*P* = 0.0065) and of 48% (*P* = 0.0011), respectively (Table 2). The incidence of contralateral breast tumors, of regional and distant relapse, and OS demonstrated no significant difference within the two groups.

In the multivariate analysis of the prognostic factors, young age (≤40 years), symptomatically detected DCIS, high nuclear grade (G2-3), solid/comedo or cribriform growth pattern, and the absence of free margins were associated with an increased risk of LR. Similarly to the findings of the NSABP B-17 trial, RT reduced the risk of LR in all prognostic subgroups considered.

The third study, the Swedish SweDCIS [21], was conducted between September 1987 and December 1999; 1046 patients out of 1067 randomized women were eligible to the statistical evaluation, with a mean followup of 8.4 years. In 823/1046 cases (78.7%), the DCIS was discovered in a mammographic screening (Table 1).

In the RT arm, treatment could be given either continuously (total dose: 50 Gy, 25 fractions) or in a split-course schedule (54 Gy given in two series with a gap of two weeks), which was administered in less than 50 cases; no RT boost was given to the tumor bed. The protocol did not require pathologically negative margins, thus in 11% of the cases, the margins were positive and in 9% they were unknown. The difference of the LR rate was significant: 27.1% in the control group compared to 12.1% in the other one. The invasive LR were reduced from 12.3% to 7.2% and the DCIS LR from 14.8% to 4.9% with RT (Table 2). The incidence of contralateral events, metastasis, and death due to breast carcinoma did not present significant differences within the two groups.

Considering the main prognostic factors, a correlation between RT effectiveness and age was noted (*P* = 0.07), more evident for women over 60 years (risk reduction of 18%) [20].

In a previous study [22] that had investigated the histopathologic risk factors for LR by a slide revision of 2 cohort cases from the trial, high nuclear grade and necrosis were associated to a major local relapse risk. RT has conferred a reduction of relapse risk in all prognostic subgroups.

The fourth study, the English, Australian and New Zealand UK/ANZ DCIS trial [23], was conducted from May 1990 in Great Britain and September 1991 in Australia and New Zealand to August 1998 (Table 1). The protocol required the complete excision of the lesion, the radiography of the surgical specimen, and the presence of free microscopic margins. In the two arms with RT (RT, RT + TAM), the total dose was of 50 Gy in 25 fractions, without boost on the tumor bed; in the two hormonal-therapy arms (TAM, TAM + RT), the TAM dose was of 20 mg/die for 5 years.

The recent update of the study [24] has a median followup of 12.7 years, and it contains 1694 patients, 1030 of which were assigned to randomization for RT. In such a group, the incidence of ipsilateral events was of 19.4% in the control arm and of 7.1% in the RT arm (*P* < 0.0001), with, respectively, 9.1% and 3.3% of invasive carcinomas and 9.7% and 3.8% of noninvasive ones; the incidence of contralateral tumors was of 4.1% in the control arm and of 3.3% in the RT arm (*P* = 0.6) (Table 2). Overall, there was no significant difference in the death rate within the different subgroups.

It was noted an increase in cardiovascular deaths within the patients treated with RT, with or without TAM (*P* = 0.008), although the numbers were small. Death due to breast carcinoma proved to be higher within the patients that received hormonal treatment, but the difference was not significant [24].

### 3.1. The Meta-Analyses of Randomized Trials

The first meta-analysis of the four randomized trials was published by Viani et al. in 2007 [25].

The overall statistical evaluation of 3665 patients pointed out a reduction in LR risk (either invasive or DCIS) of approximately 60% with the addition of adjuvant RT, compared to excision alone. Such reduction in risk was more evident in the cases with high nuclear grade and positive margins, even though it was not possible to identify a subgroup of women with low LR risk who did not need to be treated with RT. Within the two groups, no differences appeared between the incidence of distant metastasis and the OS rate. The probability of contralateral breast carcinoma proved to be 1.53 times higher in the RT arm (3.85% versus 2.5%, *P* = 0.03). In the subsequent meta-analyses, which have examined the data of the four trials after a longer follow-up period, such difference was minimal and did not prove to be statistically significant.

In 2009, Goodwin et al. [26] published a systematic review and a meta-analysis of the four trials; a more detailed version was published in the Cochrane Library [27].

From the statistical analysis, conducted on 3925 women, a significant reduction of ipsilateral events with adjuvant RT resulted (HR = 0.49; 95% CI 0.41–0.58, *P* < 0.00001). The authors investigated the role of breast irradiation in relation to some prognostic parameters: margin status, age (>50 versus <50 years), and presence of comedo necrosis; in all the analyzed prognostic subgroups the contribution of RT was significant.

In very few cases, severe cardiovascular toxicity or the appearance of a second primary tumor was observed, but with very similar numbers within the two groups. The median followup of the trials included in the meta-analysis varied from 4.4 to 10.5 years; the authors hypothesized that with a longer followup, an increase of RT late toxicity could appear [26].

In 2010, the meta-analysis of the Early Breast Cancer Trialists' Collaborative Group (EBCTCG) was published [28]. The statistical analysis showed that RT is able to reduce the absolute risk of ipsilateral events of 15.2% at 10 years (12.9% versus 28.1%, logrank 2*P* < 0.00001) and that its effectiveness does not differ significantly in relation to age, extension of the surgical procedure, association with TAM, diagnostic method, status of the margins, focality, nuclear grade, presence of comedo necrosis, architectonic pattern, and dimension of the tumor. Regarding age, a reduction of the ipsilateral events was noted in women ≥50 years of age.

The authors examined a subgroup of 291 cases out of the meta-analysis with low risk (tumor dimension ≤20 mm, negative margins and grade 1); even in such a very favourable subgroup they confirmed a significant reduction in the 10-year risk of ipsilateral events of 18.0% with RT (logrank 2*P* = 0.002).

## 4. Prognostic Factors

Actually, there is no evidence from observational studies and clinical trials that BCS plus RT is more or less effective than BCS without RT in the presence or absence of particular adverse prognostic factors, with RT being more effective in all subsets of patients.

BCS alone, even though not detrimental in terms of survival, is a treatment at high risk of LR.

Eastern Cooperative Oncology Group (ECOG) reported a registration study (ECOG E5194) aimed to prospectively identify favorable patients with DCIS treatable with local excision alone [29]. With a median followup of 6.2 years, the 7-year rate of LR was 10.5% for the 565 patients with low- or intermediate-grade DCIS; with a median followup of 6.7 years, the 7-year rate of LR was 18.0% for the 105 patients with high-grade DCIS. On multivariate analysis, no variable was significantly associated with LR.

Another study has demonstrated similar findings for a cohort of patients treated with BCS alone [30].

Wong et al. [36] reported on 158 patients treated with wide excision alone, with a minimum negative margin width of 1.0 cm. The 5-year rate of LR was 12%; the study, therefore, was closed early because of the negative results.

### 4.1. Clinical Factors

The major clinical factors associated with an increased risk of LR following breast-conserving treatment for DCIS are symptomatic presentation and young patient age at diagnosis [37].

### 4.2. Pathologic Factors

The most frequently reported factors associated with a higher risk of LR are high nuclear grade, comedo necrosis, and larger tumor size.

In the NSABP-B17 trial, the presence of comedo necrosis was associated with a higher risk of LR, limited, however, to the group of patients treated with excision alone [18]. In that group, LR rate at 8 years was 40% for cases with moderate or marked comedo necrosis compared to 23% for patients without it.

Data published by Silverstein et al. [10] showed that margin widths of 10 mm or more have a risk of LR unaffected by nuclear grade and addition of RT. In contrast, these factors remained significant in the group of patients with small margin widths [38].

The impact of DCIS grade on LR risk appears to be related to the length of followup, as emphasized in the study of Solin et al. [5]. In this study, comedo architecture and nuclear grade 3 had a significantly higher 5-year LR rate; the difference, however, was no longer statistically significant at 10 years.

These data suggest that risk factors for noninvasive and invasive LR may not be identical, that the analysis of the combination of these events into a single group may obscure important differences and that the biological basis for noninvasive and invasive LR may be different.

### 4.3. Margins

Multiple retrospective studies and some clinical prospective trials have shown that achieving pathologically confirmed negative margins is associated with a decreased rate of LR. The evaluation of the involvement of the margins of excision is one of the few clinical variables that can be controlled even though the definition of a negative margin varies from study to study (1, 2, 5, or even 10 mm).

Dunne et al. [39] reported a meta-analysis of 4660 patients treated with BCS + RT from 22 studies with data on margins of resection. The odds ratio for LR was 2.56 (*P* < 0.05), 2.89 (*P* < 0.05), and 1.51 (*P* > 0.05) for a minimum negative margin width of no tumor cells, 1 mm and 2 mm on ink, respectively.

A minimum negative margin width of 2 mm was considered appropriate in the setting of adding RT after lumpectomy. On the contrary, a minimum negative margin width of 10 mm has been recommended when using lumpectomy alone [38].

In some series, a small number of patients with close or positive margins is reported. In these cases with focally close or positive margin of resection, reexcision is the preferred next step. If a reexcision cannot be performed, definitive irradiation can be delivered with a slightly higher risk of local failure. The excess risk of LR in this setting is estimated of approximately 5%–7% [39, 40].

## 5. Salvage Treatment for Local Recurrence

Since DCIS is associated with a low rate of mortality, analyses of the success of treatment should focus on recurrence.

One of the arguments advanced in favor of omitting radiation after BCS at the time of initial presentation is the hypothetical ability to repeat a salvage breast conservative treatment. However, few data have been reported on this argument. Salvage mastectomy is frequently indicated following LR, particularly when reexcision would be cosmetically unacceptable, or when an adjuvant RT was previously performed.

The rate of salvage breast conservation is only 42%–52% after primary BCS and some patients can reject secondary breast conservation in favor of mastectomy. Thus, preventing LR by adding RT at the time of initial treatment may be a more important long-term strategy.

A dedicated study [41] reported 90 patients with local or local-regional recurrence as the site of first failure. Salvage surgery was mastectomy for 76/90 (84%) patients. The median followup was 5.5 years after salvage treatment; 10-year OS and CSS were 83% and 95%, respectively; 10-year rate of freedom from distant metastases was 91%.

Local and local-regional recurrences can be salvaged with high rates of survival and freedom from distant metastases. Careful followup is warranted for the early detection of potentially salvageable recurrences.

## 6. Particular Aspects of DCIS Irradiation

### 6.1. Role of the Boost

The advantage of an additional boost to the tumor bed after BCS and whole breast irradiation (WBI) for invasive breast cancer has been confirmed by controlled clinical trials [42, 43] and is now a standard of care. Whether this is applicable to patients with pure ductal carcinoma in situ (DCIS) is unclear.

The importance of a boost in the local control of DCIS has been examined in 6 retrospective studies [31–35, 44] (Table 3), while no prospective randomized study has been published so far on this issue.

In two trials, the role of the addition of a boost is studied: the BIG 3-07/TROG 07.01 and the BONBIS multicenter study [45, 46]. In both trials the accrual of the patients is still ongoing. 

Of particular interest are the results of the international multicenter retrospective study by Omlin et al. [31]: 373 patients ≤45 years old were treated with BCS only (15%), BCS followed by WBI (45%), or BCS and WBI followed by a boost on the tumor bed (40%). The authors observed a progressive increase of local relapse-free survival at 10 years, starting from BCS without WBI (46%), to WBI without boost (72%), to WBI followed by boost (86%) (*P* < 0.0001).

In the multivariate analysis, margin state and RT dose resulted as independent predictive factors of local relapse-free survival; the major advantage correlated to the delivery of the boost was noticed in young women (≤39 years).

Considering the limits of this study (retrospective design, with a very long accrual period of 26 years and lacking a centralized revision of tumor-sample histology), and while waiting for the results of the randomized clinical trials in course, it would be appropriate to consider a boost after WBI for women aged 45–50 or younger.

### 6.2. Hypofractionated RT

Recently, a considerable interest for hypofractionated RT schedules was noted, either in the form of whole- or partial-breast treatment.

The considerable duration of the whole treatment with conventional RT (from 5 to 6.5 weeks), associated with the distance from the patients home to the Radiotherapy Centers and the long waiting lists, represents matter-of-fact criticality factors of standard breast RT.

#### 6.2.1. Hypofractionated WBI

The efficacy of hypofractionated WBI, in the treatment of invasive breast cancer after conservative surgery, has been confirmed by retrospective studies and by some recent randomized trials, which account excellent results both in terms of local-regional control and cosmetic outcome [47].

As long as DCIS is concerned, so far only two prospective phase I and II studies have been published [48, 49].

The study of Constantine et al. [49] is the only one that included exclusively patients with mammographically detected pure DCIS (59 cases) treated with hypofractionated WBI, for a total dose of 42 Gy in 15 fractions. With a still limited followup (36 months) no LRs or contralateral tumors were found.

The study of Freedman et al. [48] examined together women with early invasive carcinoma and with DCIS. The patients underwent a treatment of the whole breast with IMRT, at a total dose of 45 Gy in 20 fractions and a concomitant boost on the tumor bed to a total dose of 56 Gy (2.8 Gy/fr. in 20 fractions). To-date, only data relative to acute toxicity and cosmetic results have been published.

Also some retrospective studies [35, 44, 50, 51] show optimal results in terms of local control in DCIS with hypofractionated treatment.

No phase III prospective study has been published until now; in 2007, the above-mentioned international multicenter BIG 3-07/TROG 07.01 trial was initiated, and it confronted both the conventional RT schedule versus the hypofractionated one, and the boost versus no-boost delivery [45, 46]. The patients' accrual has not been closed so far: some years are needed before the results can be published.

#### 6.2.2. Partial Breast Irradiation (PBI)

PBI has been widely proposed as the treatment for early-stage invasive breast cancer; options include brachytherapy techniques (using either the interstitial catheters or the intracavitary device MammoSite), external-beam RT (3D-CRT or IMRT), and intraoperative radiotherapy.

It seems more controversial when it is used to treat DCIS, based on the knowledge of the growth pattern of this tumor within the complex ductal-lobular system of the breast [52].

According to a recent Consensus Statement of the ASTRO (American Society for Radiation Oncology) [53], which has established the criteria for the inclusion of PBI, outside the clinical trials, the partial irradiation for pure DCIS is to be evaluated with caution if the tumor diameter is ≤3 cm and is not indicated if the diameter is >3 cm. Therefore, few studies have documented the efficacy of PBI in treating DCIS so far [54].

The American Society of Breast Surgeons (ASBS) has recently published an update on the DCIS case studies included in the MammoSite Registry Trial, that represents the widest prospective database published until now [55]. For 194 patients, with a median followup of 54.4 months, the 5-year actuarial LR rate is of 3.39% and the cosmetic results are favorable in 92% of the cases.

In 2005, a phase III prospective, randomized, multicenter study, NSABP B-39/RTOG 0413, was started [45, 54]. It compared WBI to PBI. Patients with DCIS or with stage I or II invasive carcinoma (*T* ≤ 3 cm), N− or N+ (≤3 N+), are being treated with lumpectomy and then randomized to either WBI (±boost) or PBI with one of the 3 following techniques: interstitial multicatheter brachytherapy (34 Gy–3.4 Gy/fr, BID), brachytherapy with MammoSite (34 Gy–3.4 Gy/fr, BID), 3D-CRT (38.5 Gy–3.85 Gy/fr, BID).

The results of this trial will be very important in finding out the long-term efficacy of PBI, both for invasive carcinoma and DCIS.

It should be noted that PBI does not represent a therapeutic standard, neither for invasive carcinoma, nor for DCIS; the randomized studies in progress will have to evaluate the relapse risk, the cosmetic results, and long-term toxicity.

## 7. Conclusions

Randomized trials provide consistent evidence that DCIS treated with breast-conserving surgery plus radiation compared to BCS alone results in a reduction of noninvasive LR and of invasive LR by approximately 50%. As breast cancer specific survival after DCIS is uniformly excellent, the major measure of treatment effectiveness has generally been the LR rate.

Subset analyses of randomized controlled trials do not point out to differential effectiveness of surgery versus RT in the presence of some adverse prognostic factors. This suggests that treatment alone may not eliminate the adverse prognosis. However, it also suggests that for patients with adverse prognostic features, treatment may be particularly important.

Studies of new irradiation modalities (PBI, hypofractionation) in DCIS patients are currently ongoing and deserve further attention.

## Figures and Tables

**Table 1:** Characteristics of the prospective randomized trials.

	NSABP-B17	EORTC 10853	SweDCIS	UK/ANZ DCIS
Entry dates	1985–1990	1986–1996	1987–1999	1990–1998
Pts. randomized	818	1010	1067	1030
CS	405	503	533	508
CS + RT	413	510	534	522
Mammographic detection	80.4%	71%	78.7%	NS
Central pathological review	76%	85%	26%	0%
Negative margins required	Yes	Yes	No	Yes
Margins free	78%	83%	80%	100%
RT dose	50 Gy/25 fr.	50 Gy/25 fr.	50–54 Gy/25–27 fr.	50 Gy/25 fr.
Boost	10 Gy/5 fr. (9% of pts.)	10 Gy/5 fr. (5% of pts.)	NR	NR

Legend: Pts: patients; NS: not specified; NR: not recommended; RT: radiotherapy; CS: conservative surgery; fr.: fractions.

**Table 2:** Comparison of breast cancer events in prospective randomized trials.

	NSABP-B17 (17.25 yrs. median FU)		EORTC 10853 (10.5 yrs. median FU)		SweDCIS (8.4 yrs. mean FU)		UK/ANZ DCIS (12.7 yrs. median FU)	
	CS %	CS + RT%	CS%	CS + RT%	CS%	CS + RT%	CS%	CS + RT%
Ipsilateral events								
Total	35.0	19.8	26.0	15.0	27.1	12.1	19.4	7.1
Invasive	19.6	10.7	13.0	8.0	12.3	7.2	9.1	3.3
In situ	15.4	9.0	14.0	7.0	14.8	4.9	9.7	3.8
Contralateral events	7.9	9.3	4.0	8.0	5.9	6.5	4.1	3.3
OS%	86*	87*	95	95	90**	92**	97.9^#^	96.2^#^

*At 12 yrs. followup.

**Breast cancer deaths and other deaths are reported in the study.

^#^All trial participants included.

Legend: yrs.: years; FU: followup; OS: overall survival; RT: radiotherapy; CS: conservative surgery.

**Table 3:** The influence of the boost on local control.

Studies	N. of pts.	Median age (y)	Positive margins %	Necrosis %	Median FU (months)	LR %
Omlin et al. [31]						
Boost	150	41*	7	32	72	14**
No boost	166		4	41		28**
Yerushalmi et al. [32]						
Boost	20	58	/	/	81	15
No boost	55	/	/	/		12.7
Julian et al. [33]						
Boost	692	53	21	52	168	13.8
No boost	877		15	45		14.3
Monteau et al. [34]						
Boost	147	53	50	60	89	9.3^*∧*^
No boost	55		74	64		9.6^*∧*^
Wai et al. [35]						
Boost	144	56	29	46	112	9*
No boost	338	55	12	55		6*
Wong et al. [44]						
Boost	79	58	5	56	46	0
No boost	121		0.8	49		6

*All < 45 years, **10-year LR, ^*∧*^7-year LR, ^#^Reexcision for close (<2 mm) margins.

Legend: pts.: patients; (y): years; FU: follow up; LR: local recurrence.
